# Canadian Older Adults’ Intention to Use an Electronic Decision Aid for Housing Decisions: Cross-sectional Web-Based Survey

**DOI:** 10.2196/43106

**Published:** 2023-01-18

**Authors:** Maya Fakhfakh, Virginie Blanchette, Karine V Plourde, Souleymane Gadio, Marie Elf, C Allyson Jones, Louise Meijering, Anik Giguère, France Légaré

**Affiliations:** 1 Department of Social and Preventive Medicine, Université Laval Quebec City, QC Canada; 2 VITAM – Centre de recherche en santé durable, Centre intégré universitaire de santé et services sociaux de la Capitale-Nationale Quebec City, QC Canada; 3 Department of Human Kinetic and Podiatric Medicine at Université du Québec à Trois-Rivières Trois-Rivières, QC Canada; 4 School of Health and Welfare, Dalarna University, Falun Dalarna Sweden; 5 Faculty of Rehabilitation Medicine University of Alberta Edmondon, AB Canada; 6 Population Research Centre, Faculty of Spatial Sciences, University of Groningen Groningen Netherlands; 7 Department of Family Medicine and Emergency Medicine, Université Laval Quebec City, QC Canada

**Keywords:** aged, intention, decision aid, decision support techniques, housing, unified theory of acceptance and use of technology, UTAUT, information technology, internet, shared decision-making

## Abstract

**Background:**

Older adults with disabilities such as loss of autonomy face the decision of whether to stay at home or move to a health care facility such as a nursing home. Therefore, they may need support for this difficult decision.

**Objective:**

We assessed the intention of Canadian older adults to use an electronic decision aid (eDA) to make housing decisions and identified the factors that influenced their intention.

**Methods:**

We conducted a cross-sectional study using a web-based survey targeting older adults across 10 Canadian provinces and 3 territories. We included respondents from a web-based panel who were aged ≥65 years, understood English or French, had access to an electronic device with an internet connection, and had made a housing decision over the past few months or were planning to make a decision in the coming year. We based the web-based survey on the Unified Theory of Acceptance and Use of Technology (UTAUT). We adapted 17 UTAUT items to measure respondents’ intention to use the eDA for housing decisions, as well as items measuring 4 intention constructs (performance expectancy, effort expectancy, social influence, and facilitating conditions). We also assessed eHealth literacy using both subjective and objective scales. We used descriptive statistics and multivariable linear regression analyses to identify the factors influencing the intention to use the eDA.

**Results:**

Of the 11,972 invited panelists, 1176 (9.82%) met the eligibility criteria, and 1000 (85.03%) respondents completed the survey. The mean age was 72.5 (SD 5.59) years. Most respondents were male (548/1000, 54.8%), White (906/1000, 90.6%), English speakers (629/1000, 62.9%), and lived in Ontario or Quebec (628/1000, 62.8%) and in urban areas (850/1000, 85%). The mean scores were 27.8 (SD 5.88) out of 40 for subjective eHealth literacy and 3.00 (SD 0.97) out of 5 for objective eHealth literacy. In our sample, the intention score was 4.74 (SD 1.7) out of 7. The mean scores of intention constructs out of 7 were 5.63 (SD 1.28) for facilitating conditions, 4.94 (SD 1.48) for performance expectancy, 5.61 (SD 1.35) for effort expectancy, and 4.76 (SD 1.59) for social influence. In the final model, the factors associated with intention included mother tongue (β=.30; *P*<.001), objective eHealth literacy (β=–.06; *P*=.03), performance expectancy (β=.55; *P*<.001), social influence (β=.37; *P*<.001), and facilitating conditions (β=.15; *P*<.001).

**Conclusions:**

Findings from this pan-Canadian web-based survey on Canadian older adults suggest that their intention to use the eDA to make housing decisions is similar to the findings in other studies using UTAUT. The factors identified as influencing intention were mother tongue, objective eHealth literacy, performance expectancy, social influence, and facilitating conditions. These will guide future strategies for the implementation of the eDA.

## Introduction

### Background

As in many other countries, older adults in Canada (ie, persons aged ≥65 years) are a rapidly growing segment of the population [1]. In Canada, the number of older adults increased by 18.3% between 2016 and 2021 to reach 7 million [2]. By 2030, it is estimated that older adults will account for 23% of the Canadian population [1]. As adults age, they are more likely to experience disabilities, leading to loss of autonomy [3]. For instance, in Canada, 19.5% of older adults reported that their health was perceived to be somewhat worse or much worse than 1 year ago and 16.5% had to receive assistance from family, friends, or neighbors for a health problem or disability. On the basis of the Instrumental and Basic Activities of Daily Living Classification, 93.9% of Canadian older adults experience a mild loss of autonomy [4].

To manage loss of autonomy, meet health care and social services needs, and ensure their safety and well-being, many Canadian older adults consider receiving home care, which typically includes nursing care, therapy (physical, occupational, and speech-language), and medical and social services [5]. Others consider assisted living or moving to residential health care facilities, such as nursing homes [6-8]. In this paper, all decisions about whether to stay home and age safely in place or to move out to a residential health care facility are referred to as “housing decisions” [9].

In Canada, housing decisions are considered the most frequent and difficult decisions for older adults receiving home care as well as for their caregivers [10,11]. The Ottawa Decision Framework identifies the factors influencing decisions as, in general, inadequate support and resources (or “decisional needs”) as well as personal characteristics such as gender, education, and ethnicity. In the context of housing decisions specifically, studies have shown that besides loss of autonomy, older adults in Canada consider moving for a variety of other reasons, such as caregivers’ opinions, proximity of services or relatives’ support, financial resources, and feelings of insecurity or fear at home [8,11]. One Canadian study showed that gender, age, household income, province, driving status, whether the current home met the older adults’ needs, and unmet heavy cleaning needs were all important influences on decisions to relocate [12].

Housing decisions and transitioning to long-term care can be experienced differently, depending on the sociocultural context. For instance, in Western cultures, some residential care facilities try to create a homelike atmosphere by allowing older adults to bring their furniture, pets, and family pictures to help ease the transition [13]. However, a meta-analysis showed that in the United States and Canada, older adults still experience transitioning to long-term care as a loss that requires a mourning process before coming to peace with the decision [14].

Amid the COVID-19 pandemic, housing decisions have become not only more frequent but also more painful and complicated for older Canadians [15]. The decision to relocate appears fraught with danger as long-term care homes and seniors’ residences were among the hardest-hit facilities in Canada in 2020 during COVID-19. Residents in nursing and senior homes accounted for >80% of all reported COVID-19 deaths [16].

To help older adults make informed decisions regarding the most appropriate housing option, shared decision-making (SDM) is advocated. SDM is the process of making a health care choice that involves patients, their relatives or family or both, and one or more health care professionals [17]. SDM is particularly appropriate for preference-sensitive decisions. The best housing decisions reflect older adults’ personal values and preferences, as well as those of their relatives [7,18]. As the need for self-management is increased by the cumulative effects of long-term conditions in older adults, SDM becomes more relevant, especially when it comes to housing. Besides, there is growing evidence that older adults and their caregivers would like to be more involved in decision-making [19]. Decision aids (DAs) can be used to facilitate SDM. DAs are evidence-based tools that support older adults in defining decisions, provide information regarding different options and outcomes, and help clarify personal values and priorities [20].

eHealth refers to health services and information delivered through the internet and related technologies [21]. According to recent studies, eHealth can empower older adults to manage their health by providing more accessible health information through educational and supportive web platforms and connection with others with shared health interests [22-25]. The use of eHealth has increased significantly since COVID-19. The accelerated digital transformation that has occurred [24] has encouraged older adults to use internet services to meet their needs in several aspects of life (eg, web-based groceries, web-based shopping, and health appointments). Because rapidly changing health conditions can quickly overtake older adults’ housing decisions [9], they need easily accessible web-based tools that can be updated to help them obtain the information they need to participate actively in SDM.

In a clinical setting, a DA is usually presented before the clinical encounter to prepare the patient for SDM with a health professional or during the encounter to prepare for a subsequent encounter. Few health professionals have the time to work through a DA with the patient and come to a conclusion on the spot [26]. We designed an electronic version of a paper-based DA [27,28] (electronic decision aid [eDA]) to help older adults choose the most appropriate housing option for them. Older adults can use the eDA alone or with their families to prepare for SDM with a health professional. The eDA can also be used in SDM training for health care professionals to help them understand the practical steps involved in the SDM process. The paper-based DA is already available on the website of the Canada Research Chair in SDM and Knowledge Translation [28]. The eDA will also be made available free on this website. Brief details of the conversion are provided in Multimedia Appendix 1 [7]. We also plan to register it on the Ottawa Decision Aids website [20] and will suggest that partnering organizations provide hyperlinks to the eDA (eg, Fédération de l’Âge d'Or du Québec and L’Appui for caregivers).

### Objective

We hypothesized that older adults would find the eDA to be useful. To our knowledge, no study has yet investigated whether older adults would be willing to use the eDA for housing decisions. Therefore, our aim was to assess Canadian older adults’ intention to use the eDA to make housing decisions and to identify the factors influencing their intention to use it.

## Methods

### Study Design

We conducted a cross-sectional web-based survey across Canada (including the 10 provinces and 3 territories) with older adults who had either made a housing decision in the past 12 months or were planning to make a housing decision the next year. We used the consensus-based Checklist for Reporting of Survey Studies (CROSS) to guide the reporting of our results [29] (Multimedia Appendix 2). This project is part of COORDINATEs (Technology to Support Decision Making About Aging at Home), an international study (Canada, Sweden, and the Netherlands), whose objective is to use technology to help older adults stay at home as long as possible in a safe manner and to assist them in making informed decisions about aging at home [9].

### Ethics Approval

This project was approved by the Ethical Review Board of the Integrated University Health and Social Services Centre of the Capitale-Nationale, Quebec, Canada (#MP-13-2019-1519, 2019-1519_SPPL).

### Theoretical Framework to Guide the Survey Development

**Figure 1:**
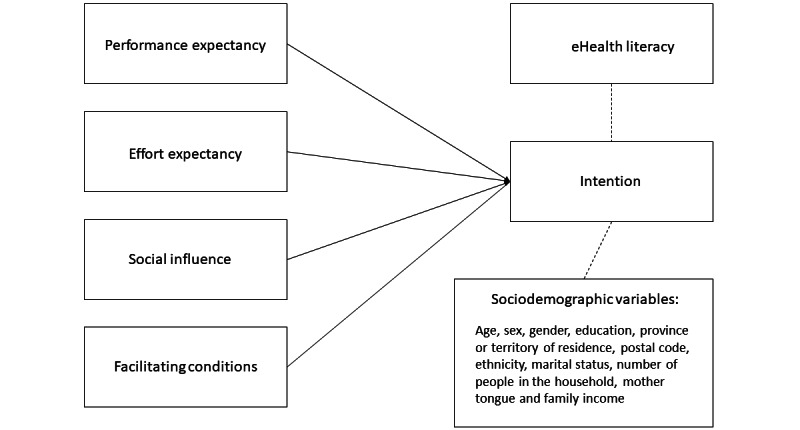
Adapted version of Unified Theory of Acceptance and Use of Technology.

Health-related behaviors are correlated with intention, which is defined as an individual’s planned and rationalized decision to perform the behavior [30]. A meta-analysis of 10 meta-analyses showed that intention explained nearly one-third of the variance in behavior [31]. Using eHealth interventions to improve one’s health first requires acceptance of technology and then the intention to use it. Several theoretical models have studied the intention (and its determinants) to use technology in health care. The Unified Theory of Acceptance and Use of Technology (UTAUT) is among the most widely used theoretical models for studying the intention to use technology in health care [32]. Developed by Venkatesh et al [32], UTAUT is an extension of several theoretical models that preceded it, such as the Technology Acceptance Model [33] and Ajzen’s Theory of Planned Behavior [34]. UTAUT explains that behavioral intention to use technology is based on four constructs: (1) performance expectancy, defined as the degree to which individuals believe that using the system will help them gain advantages; (2) effort expectancy, defined as the degree of ease associated with the use of the system; (3) social influence, defined as the degree to which individuals perceive that their family, friends, and society in general would approve of them using the new system; and (4) facilitating conditions, defined as the degree to which an individual believes that an organizational and technical infrastructure exists to support the use of the system [32]. To achieve the aim of this study, we adapted this framework by adding variables that could have a direct influence on the intention to use the eDA. Therefore, we measured sociodemographic variables as well as eHealth literacy, defined as “the use of emerging information and communication technology to improve or enable health and health care” [35]. eHealth literacy combines different dimensions of literacy skills (traditional literacy, health literacy, information literacy, scientific literacy, media literacy, and computer literacy; Figure 1) [36].

### Respondents and Recruitment

Respondents were eligible if they were Canadian adults aged ≥65 years, understood English or French, had access to an electronic device with an internet connection, and had made a housing decision in the past few months or were planning to make one in the coming year.

We recruited respondents through Leger Marketing, a market research and analytics company in Montreal, Canada. Leger Marketing is the largest private Canadian web-based panel (400,000 individuals) and claims to be representative of the entire population [37]. It adopts a hybrid recruitment approach using traditional and mobile telephone methodologies through call centers, panel websites, and social media. It also updates panelists’ profiles every 6 months and controls the accuracy and quality of participants’ answers using validation techniques (eg, username and strict restrictions on passwords, deduplication with existing panel, and simultaneous recruitment campaigns) from the beginning of the registration process [37]. Leger Marketing selected our sample from its panel of 400,000 individuals using a nonprobability sampling method. An electronic invitation was sent to 11,972 older adults to complete the closed survey. The sampling methods used were representative of the general Canadian population in terms of age, gender, region, and socioeconomic status. Upon obtaining consent, eligible respondents were able to complete the survey. The survey was conducted on a voluntary basis and compensation was offered to respondents in the form of Leger Points, which are redeemable for cash or gift cards.

Each respondent from the web-based panel received a personalized email invitation containing a unique URL link to access the nonopen survey. Respondents were then asked to answer the first questions about language preference, province or territory of residence, and eligibility. Leger Marketing sent reminders via email once a week, until the survey was closed. As respondents logged into the survey using their panel membership account, we had a unique response per member because it was not possible for the same member to have multiple submissions.

A minimum of 829 participants were required. The sample size was estimated using the central limit theorem formula [38]. This formula provides the recommended sample size to estimate the true population mean with the required margin of error and level of confidence. To determine the sample size, a similar study by Yousef et al [39] was chosen to estimate the SD of the mean intention score in the population (SD 0.56). The survey was closed once 1000 respondents had completed the survey. Recruitment took 4 weeks (April 5 to May 2, 2022).

### Data Collection

Because no validated instruments that assess older adults’ intention to use an eDA for housing decisions have been identified, we created a self-administered questionnaire based on the adapted UTAUT items. We measured our main outcome, intention, and its 4 determining constructs using the 17 UTAUT-based items. Each UTAUT construct (intention, performance expectancy, effort expectancy, social influence, and facilitating conditions) was measured using 3 or 4 items. Respondents indicated their agreement or disagreement levels with the corresponding items on a 7-point Likert scale ranging from 1 (*strongly disagree*) to 7 (*strongly agree*). We calculated the final scores for each construct by averaging the scores of its corresponding items. In our UTAUT-based questionnaire, we replaced the word “technology” with “the web-based decision aid” and adapted the wording of each item to the context of our study. The UTAUT has good internal consistency and convergent and discriminant validity [32]. Cronbach α indicated good reliability of the multi-items measuring each construct (α range .9-.95).

The survey also collected sociodemographic characteristics (ie, age, sex, gender, education, province or territory of residence, postal code, ethnicity, marital status, number of people in the household, mother tongue, and family income) using items based on Statistics Canada’s 2021 census questionnaire [40].

We evaluated eHealth literacy using 2 scales. The first was the Electronic Health Literacy Scale (eHeals) [41], a validated scale that measures respondents’ self-rated literacy level (referred to as “subjective scale”). eHeals was developed by Norman and Skinner [41] and is regarded as the “gold standard” for measuring eHealth literacy [41]. It is a validated 8-item scale with high internal consistency [42]. For each of the 8 items, respondents expressed their agreement or disagreement on a 5-point Likert scale (1=*strongly disagree* to 5=*strongly agree*), with higher scores reflecting better literacy skills. The eHeals scale generated a total score ranging from 8 to 40. On the basis of the different thresholds used in the literature to better classify literacy levels [43,44], eHealth literacy was considered high if the score was ≥26. We also used the Digital Health Literacy Instrument (DHLI) [45] to evaluate eHealth literacy. The DHLI (referred to as the “objective scale”) is a measure of the actual performance of respondents when using internet web pages. The DHLI subscale, originally Dutch, measures health-related internet use skills using 7 items [45]. The DHLI consists of screenshots of web pages with questions that assess operational skills, navigation skills, evaluation of reliability, determination of relevance, information searching, addition of self-generated content, and protection of privacy [45]. For this project, we adapted the DHLI to the Canadian context. After discussion with our research team members, only 5 items were included in the questionnaire. The 2 items concerning content addition and privacy protection were not directly related to this project and were omitted. If the respondent gave the correct answer, they received a score of 1. Otherwise, they obtained a score of 0. Therefore, the maximum possible score was 5. Cronbach αs for eHeals and DHLI were, respectively, .91 and .35.

After completing the 2 eHealth literacy scales, the respondents were shown a 6-minute video vignette showing the use of the eDA in context. As mentioned by Godin et al [46], it is necessary to clearly define the targeted behavior (ie, use of the eDA for making housing decisions) before measuring the intention related to that behavior. The video was a demonstration of the SDM process regarding housing decisions and showed a situation where an autonomous older adult interacted with her caregiver who was concerned about her safety [47,48]. In the video, the older adult discussed the different housing options while using the eDA with her caregiver. All participants had to see the video to continue the survey and complete the UTAUT questions. The respondents were then asked to browse through the eDA [49]. Subsequently, UTAUT was used to evaluate respondents’ intention (and its related constructs of performance expectancy, effort expectancy, social influence, and facilitating conditions) to use the eDA [32]. On the basis of the eDA, the survey ended with questions about the process of making housing decisions using the following variables: chosen housing decision (“Which option did you choose or do you consider choosing?”), preferred housing options (“Which option do you prefer?”), reasons for considering housing options (“What are your reasons for making this decision?”), support in the decision-making process (“Who had helped you or can help you to make this decision?”), and preferred role in the decision-making process (“If you had to make this decision, how would you prefer the decision to be made?”). We treated these decision-making process variables as descriptive variables and did not include them in the multivariable analysis.

The survey was 48 web pages long, took approximately 30 minutes to complete, and consisted of 50 closed-ended questions that were not randomized and appeared in the same order for all respondents. Each page included a “next” button for moving forward and a button with a list of older adult helplines for talking to a specialist who could support them mentally or emotionally if they were uncomfortable with any of the survey questions. Respondents could not move to the next page unless they had completed all the questions on the current page. Surveys were labeled as complete only if respondents had clicked on the “finish” button located at the end of the survey. Both English and French versions were pretested with a sample of 76 respondents to identify any possible ambiguity or technical problems, validate the clarity of the questions, and estimate the average completion time. No major revisions were made following the pretest.

### Data Analysis

We determined the distribution of our population for sociodemographic variables, levels of eHealth literacy, UTAUT constructs, and decision-making process variables using descriptive statistics (means, SDs, and percentages). Because intention scores could vary between 1 and 7, we interpreted intention as a continuous variable. There is no definitive threshold for a clinically significant intention score in the literature. We used the Shapiro-Wilk test to verify whether the distribution of the dependent variable was normal.

We considered the “prefer not to answer” choice as missing data (1.8%, 18/1000) for bivariate and multivariable analyses, except for the income variable. We calculated the age of the respondents by considering their date of birth and date of survey completion. We performed a mixed linear regression model including all the independent variables, that is, age, sex, gender, education, province or territory of residence, postal code, ethnicity, marital status, number of people in the household, mother tongue, family income, eHealth literacy (objective and subjective), performance expectancy, effort expectancy, social influence, and facilitating conditions, using stepwise selection with the Bayesian Information Criterion [50]. We used an alternative variable selection approach to validate our model. We conducted a bivariate analysis using simple linear regressions on each variable to describe the associations between the dependent variable (intention to use the eDA for housing decisions) and the independent variables. Variables with *P* values <.10 were considered significant, a threshold more stringent than the usual .20. We then included the selected independent variables in a multivariable analysis model and identified the factors associated with intention. We checked collinearity using a correlation matrix of the continuous variables (age, number of people in the household, eHealth literacy, performance expectancy, social influence, and facilitating conditions; Multimedia Appendix 3). All analyses were performed using SAS (version 9.4; SAS Institute Inc) [51].

## Results

### Respondents’ Characteristics

**Figure 2:**
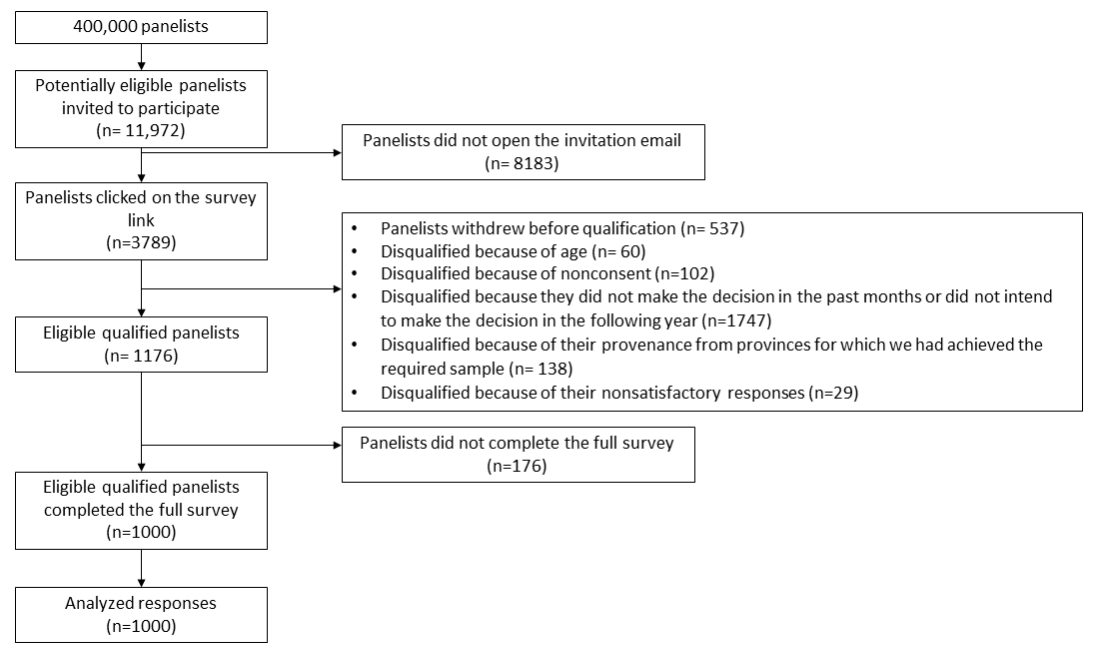
Flow of respondents.

Of the 11,972 panelists who were invited to participate, 3789 (31.65%) panelists clicked on the survey link received by email; 1176 (31.04%) met the eligibility criteria; and 1000 (85.03%) respondents completed the entire survey and were included in the analysis (Figure 2). The response rate was 31.65% (ratio of 3789 users who clicked on the survey link to 11,972 invitations sent), and the completion rate was 85.03% (ratio of 1000 users who completed the survey to 1176 eligible users who participated).

The included respondent’s characteristics are listed in Table 1. For the respondents who withdrew from the survey (14.97%, 176/1176), they had sociodemographic characteristics similar to those who fully completed the survey. Most respondents who withdrew were male (103/176, 58.5%) with a mean age of 73.9 (SD 6.0) years, White (160/176, 90.9%), living in Ontario or Quebec (101/176, 57.4%), and many were highly educated (64/176, 36.4% were university graduates). For the 1000 included respondents, the mean scores were 27.8 (SD 5.88) out of 40 for subjective eHealth literacy and 3.00 (SD 0.97) out of 5 for objective eHealth literacy. We consider that both scores represented high eHealth literacy levels. The subjective eHealth distribution was slightly skewed to the right in the direction of the highest score.

**Table 1:** Respondents’ characteristics (n=1000).

			Respondents
Age (years), mean (SD)			72.5 (5.6)
**Sex, n (%)**			
	Male	548 (54.8)	
	Female	452 (45.2)	
**Gender, n (%)**			
	Man	546 (54.6)	
	Woman	454 (45.4)	
**Level of education, n (%)**			
	A university certificate, diploma, or degree (eg, bachelor’s degree, degree in medicine, dentistry, and veterinary medicine)	420 (42)	
	A college, CEGEP^a^, or other nonuniversity certificate or diploma (other than trade certificates or diplomas)	264 (26.4)	
	A high school (secondary school) diploma or equivalent, a registered apprenticeship, or other trade certificate or diploma	286 (28.6)	
	Lower than a high school (secondary school) diploma or equivalent (eg, primary school)	25 (2.5)	
	I prefer not to answer	5 (0.5)	
**Province or territory of residence, n (%)**			
	Ontario	377 (37.7)	
	Quebec	251 (25.1)	
	Western Canada: British Columbia, Alberta, Saskatchewan, Manitoba, and Yukon	295 (29.5)	
	Eastern Canada: New Brunswick, Nova Scotia, Prince Edward Island, and Newfoundland and Labrador	77 (7.7)	
**Zone, based on postal code, n (%)**			
	Urban	850 (85)	
	Rural	141 (14.1)	
	I prefer not to answer	9 (0.9)	
**Ethnicity, n (%)**			
	White	906 (90.6)	
	Non-White	73 (7.3)	
	Indigenous peoples of North America (First Nations, Métis, or Inuk [Inuit])	18 (1.8)	
	I prefer not to answer	3 (0.3)	
**Marital status, n (%)**			
	Legally married (and not separated)	516 (51.6)	
	Divorced	152 (15.2)	
	Widowed	138 (13.8)	
	Never legally married	93 (9.3)	
	In a common-law union	81 (8.1)	
	Separated, but still legally married	19 (1.9)	
	I prefer not to answer	1 (0.1)	
Number of people in the household, mean (SD)			1.80 (0.81)
**Mother tongue, n (%)**			
	English	629 (62.9)	
	French	283 (28.3)	
	Other^b^	88 (8.8)	
	Aboriginal languages^c^	0 (0)	
**Family income,** **n (%)**			
	CAD $100,000 (US $76.923) or more	172 (17.2)	
	CAD $75,000-$99,999 (US $57.700-US $76.922)	153 (15.3)	
	CAD $50,000-$74,999 (US $38.461-US $57.692)	221 (22.1)	
	CAD $25,000-$49,999 (US $19.230-US $38.460)	262 (26.2)	
	<CAD $25,000 (US $19.230)	114 (11.4)	
	I prefer not to answer	78 (7.8)	
eHealth literacy (subjective)^d^, mean (SD)			27.8 (5.88)
eHealth literacy (objective)^e^, mean (SD)			3.00 (0.97)

^a^CEGEP: Collège d'enseignement général et professionnel.

^b^Other: Spanish, Mandarin, Arab, Cantonese, Dutch, Flemish, German, Greek, Gujarati, Hindi, Hungarian, Igbo, Indo, Italian, Lithuanian, Polish, Portuguese, Punjabi, Romanian, Russian, Serbian, Slovak, Slovenian, Tamil, Ukrainian, and Urdu.

^c^Aboriginal languages in Canada: Algonquian languages (eg, Cree, Ojibway, Innu or Montagnais, and Mi’kmaq), Inuit languages, Athabaskan languages, Salish languages, Siouan languages, Iroquoian languages, Tsimshian languages, Wakashan languages, Michif, Haida, Tlingit, and Kutenai.

^d^Sum of 8 items on a 1 to 5 Likert scale (1=strongly disagree and 5=strongly agree). Scores range from 8 to 40.

^e^Sum of 5 items (score 0 if wrong answer, score 1 if correct answer). Scores range from 0 to 5.

### Intention and UTAUT Construct Scores

The UTAUT construct scores are shown in Table 2. The mean score of older adults’ intention to use the eDA to decide about housing was 4.74 (SD 1.7) out of 7. We considered intention scores of ≥4 sufficient to assume that older adults would use eDA for housing decisions. As for the other UTAUT constructs, facilitating conditions had the highest mean score of 5.63 (SD 1.28) out of 7. Older adults seemed to believe that organizational and technical infrastructure existed to support the use of the eDA. The mean scores of performance expectancy, effort expectancy, and social influence were, respectively, 4.94 (SD 1.48), 5.61 (SD 1.35) and 4.76 (SD 1.59), that is, older adults believed that the eDA would help them to make better decisions and found it easy to use, and the eDA was approved by their relatives, so they would be more inclined to use it.

Cronbach α values are presented for each assessed construct and are a measure of internal consistency for each construct. This is considered to be a measure of scale reliability.

The intention scores associated with each decision-making process variable are listed in Table 3.

**Table 2:** Unified Theory of Acceptance and Use of Technology (UTAUT) construct scores (n=1000).

UTAUT construct^a^	Scores, mean (SD)	Cronbach α
Intention	4.74 (1.70)	.95
Performance expectancy	4.94 (1.48)	.94
Effort expectancy	5.61 (1.35)	.95
Social influence	4.76 (1.59)	.95
Facilitating conditions	5.63 (1.28)	.90

^a^Averaging the scores of the corresponding items on a Likert scale from 1 to 7. The scores range from 1 to 7.

**Table 3:** Intention scores associated with decision-making process variables (n=1000).

				Respondents, n (%)		Intention scores, mean (SD)
**Chosen housing option**						
	**Only 1 chosen option**					
		Stay in your home	736 (73.6)		4.64 (1.7)	
		Move to a family member’s home	17 (1.6)		4.29 (1.8)	
		Move to a private seniors’ residence	78 (7.8)		5.25 (1.5)	
		Move to a public residential or long-term care center	16 (1.6)		5.37 (1.7)	
	2 chosen options		88 (8.8)		4.87 (1.7)	
	3 chosen options		14 (1.4)		5.30 (1.6)	
	4 chosen options		2 (0.2)		6.00 (0.0)	
	Other option, specify		49 (4.9)		4.8 (2.0)	
**Preferred housing option**						
	Stay in your home		843 (84.3)		4.67 (1.7)	
	Move to a family member’s home		25 (2.5)		4.79 (1.6)	
	Move to a private seniors’ residence		75 (7.5)		5.2 (1.6)	
	Move to a public residential or long-term care center		17 (1.7)		5.43 (1.8)	
	Other option, specify		40 (4)		4.9 (1.9)	
**Reasons for considering housing options**						
	**Only one reason**					
		Someone else thinks you should move	26 (2.6)		4.93 (1.7)	
		You are concerned about your health	149 (14.9)		5.11 (1.5)	
		You are less able to walk or move around	44 (4.4)		3.95 (1.4)	
		You do not feel safe	8 (0.8)		5.08 (1.8)	
		You do not have enough help at home	21 (2.1)		5.23 (1.7)	
		You feel lonely	31 (3.1)		4.83 (1.6)	
		You have trouble doing your groceries, getting to the pharmacy, getting to the physician’s office, etc	28 (2.8)		4.87 (1.5)	
		Your relatives can no longer give you the support you need	22 (2.2)		5.0 (1.8)	
	More than 1 reason		253 (25.3)		5.05 (1.6)	
	Other option, specify		418 (41.8)		4.42 (1.8)	
**Support in the decision-making process**						
	Spouse		271 (27.1)		4.44 (1.8)	
	Children		181 (18.1)		4.83 (1.6)	
	Grandchildren		5 (0.5)		4.13 (2.3)	
	Other family member		53 (5.3)		4.83 (1.6)	
	Friends		46 (4.6)		4.63 (1.6)	
	Physician		26 (2.6)		4.14 (1.7)	
	Social worker		11 (1.1)		4.82 (2.2)	
	Family and friends only		211 (21.1)		4.9 (1.6)	
	Health care team only		10 (1)		6.0 (0.9)	
	Both (family, friends, and health care team)		186 (18.6)		5.02 (1.6)	
**Preferred role in the decision-making process**						
	Active (“I make the decision alone, I make the decision alone but consider the opinion of my relatives and/or health care providers, we decide together with my relatives and/or health care providers, equally”)		973 (97.3)		4.73 (1.7)	
	Passive (“My relatives and/or health care providers make the decision but consider my opinion, my relatives and/or health care providers make the decision alone”)		27 (2.7)		5.01 (1.6)	

### Factors Associated With Intention

**Figure 3:**
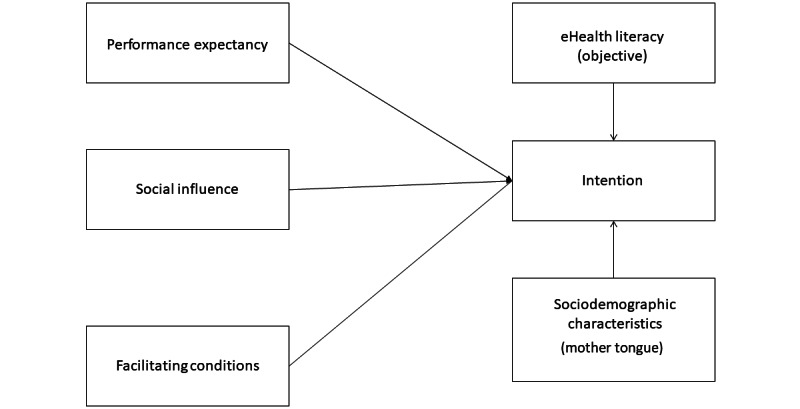
The final proposed model.

Table 4 shows factors significantly associated with intention in the multivariable model. In order of importance, these factors were performance expectancy (β=.55; *P*<.001), social influence (β=.37; *P*<.001), mother tongue (β=.30; *P*<.001), facilitating conditions (β=.15; *P*<.001), and eHealth literacy (objective) (β=−0.06; *P*=.03). On the basis of these results, we proposed a modified parsimonious UTAUT model (Figure 3). Overall, our final model explained 73.3% of the total variance of our dependent variable.

The alternative variable selection approach (ie, the selection of independent variables in the bivariate analyses using the threshold of 0.1 before conducting the multivariable analysis) resulted in the same final model. In total, 9 variables were retained in the bivariate analyses (Multimedia Appendix 4).

**Table 4:** Multivariable factors significantly associated with older adults’ intention to use the electronic decision aid.

Variable		Respondents, n (%)	β (95% CI)^a^	*P* value
**Mother tongue**				
	English (ref)^b^	629 (62.9)	N/A^c^	N/A
	French	283 (28.3)	.30 (0.17 to 0.43)	<.001
	Other	88 (8.8)	.06 (−0.11 to 0.28)	.57
eHealth literacy (objective)		1000 (100)	−0.06 (−0.1 to −0.005)	.03
Performance expectancy		1000 (100)	.55 (0.49 to 0.61)	<.001
Social influence		1000 (100)	.37 (0.32 to 0.43)	<.001
Facilitating conditions		1000 (100)	.15 (0.10 to 0.21)	<.001

^a^The estimated β for each variable and its 95% CI are presented in the table.

^b^Ref: reference category for the analysis.

^c^N/A: not applicable.

## Discussion

### Principal Findings

To the best of our knowledge, this is the first web-based survey across the 10 Canadian provinces and 3 territories to investigate older adults’ intention to use an eDA for housing decisions. The mean intention score was moderate. In addition, we found that older adults’ most chosen and preferred housing option was to stay in their homes. Most participants had multiple reasons for this preference, which were largely related to their health conditions. Older adults were mostly supported by spouses or children in making their housing decisions, and the majority preferred to play an active role in the decision-making. We also found that intention varied across Canada according to the respondents’ mother tongue. French native speakers were more likely to use the eDA for housing decisions than those with other mother tongues. In addition, objectively evaluated eHealth literacy was negatively associated with intention (ie, a lower level of eHealth literacy was associated with higher intention scores), whereas subjectively evaluated eHealth literacy was not. Finally, the UTAUT constructs of performance expectancy, social influence, and facilitating conditions were significantly and positively associated with intention. In other words, respondents with higher scores for performance expectancy, social influence, and facilitating conditions had a greater intention to use the eDA for housing decisions. These results allowed us to make the observations elaborated in the following sections.

### Interpretation and Comparison With Prior Work

First, scores representing older adults’ intention to use the eDA in this study were positive and similar to the scores in 3 studies using UTAUT model in the context of digital health care, which ranged from 2.8 to 4.42 [39,52,53]. The first study was a systematic review investigating the acceptance of web-based interventions for addressing various physical and mental health conditions among patients and health professionals [53]. The second study examined patients’ intentions to use their eHealth records [39], and the third study examined older adults’ intentions to use eHealth applications [52]. It is difficult to predict whether the intention score in our study is sufficient for older adults to adopt the targeted behavior (to use the eDA). As mentioned, there has been no definitive initiative to determine a cutoff point for clinically significant intention scores. All things considered, because there was no ceiling effect with regard to the intention score [54], suitable strategies and interventions should still be developed considering the factors influencing intention identified in our study to prompt older adults to use the eDA and thus make better informed housing decisions.

Second, our results suggest that older adults who are supported in their decision-making process by their family, friends, and health care team are more inclined to use the eDA to make housing decisions. Other studies have confirmed the importance of relatives in the decision-making process regarding housing options [10,11]. Therefore, it could be useful to add a section in the eDA to be filled in by caregivers who are involved in the decision. Comparing older adults’ and caregivers’ preferences could allow for a better understanding of each point of view and their respective needs, values, and priorities. This would better prepare older adults and their families for SDM discussions with each other and with their health professionals (eg, doctors, social workers, physiotherapists, and occupational therapists) and reduce the decisional regrets of older adults [11] and caregivers [10]. The eDA could thus be a useful tool in the implementation of an interprofessional SDM model, which stresses the importance of facilitating communication between different parties involved in the SDM process to reach common ground about the issues at stake, especially when it comes to sensitive topics such as housing decisions [55,56].

Third, contrary to our expectations, of the 11 sociodemographic variables in the study, only the mother tongue remained in the final model. Our results suggest that francophone Canadians are more inclined to use the eDA than anglophones. This might be because the province of Quebec, where most Canadian French native speakers live, has the highest percentage of older adults living in residential care in the country [57]. In 2021, 17% of Quebecers were aged ≥75 years and lived in senior residences, compared with only 5%-10% in the other provinces [57]. In addition to this cultural choice, Quebec dedicates a large share of its home care resources to tax credits, 83% of which are used to pay rent for older adults’ private long-term care residences, instead of funding public services (eg, personal support workers) to enable people to stay in their homes [58]. Owing to these budgetary choices, the proportion of older people with access to publicly funded home care services has fallen sharply over the last years. Owing to such pressures and the growing rate of older adults, housing decisions are becoming more complex than ever before in Quebec; therefore, Quebecers would see more need for a tool that supports them in their housing decisions.

Quebec is considered as a “distinct society” whose culture and social values are different from those in English Canada [59,60]. A higher willingness to use the eDA in Quebec could also be attributed to the fact that the eDA was developed by a francophone research team affiliated with Université Laval, Quebec. Although the eDA was translated into English, it was originally designed in French, and the designers of the eDA may have unintentionally reflected Quebecers’ interests and values more than those of other Canadian populations. In addition, a web-based survey assessing Canadians’ health-related decision-making processes showed that being an older adult living in the province of Quebec decreased the level of SDM experienced [61]. This could explain why Quebecers are more eager to engage in SDM and use the eDA when presented with the possibility of doing so.

Fourth, even though eHealth literacy, whether measured objectively or subjectively, was associated with intention in the bivariate analysis, only the objective measure of eHealth literacy remained in the multivariable model and seemed to have had a stronger influence on intention. This result confirms the importance of measuring eHealth literacy both objectively and subjectively. Believing oneself to have high literacy levels is not sufficient and needs to be completed with objective performance measurements, which count more in terms of assessing behavioral intentions related to health [62]. Contrary to our hypothesis, we found a negative correlation between objective eHealth literacy and older adults’ intention to use the eDA for housing decisions. The eDA was designed as a simple tool. As most respondents in our sample had high eHealth literacy scores, they might have expected a more sophisticated tool. This could explain their lower intention to use the eDA. Another possible explanation is that because eHealth literacy positively correlates with health literacy [36,63], respondents might have expected an eDA richer in content and information. Conversely, respondents with lower eHealth literacy scores were more inclined to use the eDA. This could be explained by the simplicity of the eDA. It is important to pay attention to those who have limited skills in using digital technologies. A study of low-income American older adults in 2020 showing that only half of the participants used the internet and of these, less than half had high eHealth literacy scores [44]. Older people are disproportionately affected by the “digital divide” [64]. Future research on eDAs could focus on the relationships among content, design, health literacy, objective eHealth literacy levels, and older adults’ intention to use them.

Fifth, as expected, we found that the 3 UTAUT constructs (performance expectancy, social influence, and facilitating conditions) were significantly associated with intention. In other words, the more respondents believed that the eDA would improve the quality of their decision-making, that their social circle would approve of the use of the eDA, and that they had the necessary assistance for using web-based resources, the more they intended to use the eDA to decide about housing. Only the construct effort expectancy was excluded from the final model. Our results are congruent with those of other studies related to eHealth, except for effort expectancy, which was included in their models and not in ours [65,66]. As explained by Venkatesh et al [32], if the targeted behavior has not been experienced before by older adults (ie, the use of the eDA) and if effort expectancy is not present in the model, then facilitating conditions are expected to become the main predictor of intention. This was the case in this exploratory study. Moreover, Venkatesh et al [32] stated that according to the various models on which his theory is based, performance expectancy is the strongest predictor of intention of all the constructs, which was confirmed in our study. In contrast with our findings, de Veer et al [52] excluded social influence from their final model, although they found that family and friends influenced the intention to use eHealth. This might be because we operationalized social influence differently; de Veer et al [52] used only 1 item to measure social influence, whereas we used 3. Nonetheless, we too observed that respondents with higher-than-average intention scores benefited from social support (ie, family, friends, and health care team) in their decision-making process (Table 3).

Finally, our findings suggest that UTAUT constructs and behavior change methods [67] could be used to design strategies focusing on facilitating conditions and social influence that would enhance older adults’ intention to use the eDA. For example, health and social care workers could be mobilized to promote the use of the eDA across different health care settings, in residential care facilities or when providing homecare services [67]. As also suggested by Bartholomew et al [67], mobilizing persuasive communication strategies and social networks could be helpful when disseminating the eDA. Members of social networks (eg, family members and relatives, caregivers, peers, and health care professionals) could help inexperienced older adults use eHealth resources [67,68].

### Strengths and Limitations

The strength of our study was that this was a rigorous theory-based analysis of the intentions of older adults across Canada, a country that stretches 4700 miles coast to coast, to better support them in making one of their most difficult decisions. Furthermore, Leger Marketing, the survey firm, balanced our recruited sample across age, sex, gender, and socioeconomic status. In addition, the response rate in our study (3789/11,972, 31.65%) was higher than the average for web surveys, which usually ranges from 10% to 20% [69-71], and higher than the average response rate for the Leger panel.

Our study has a few limitations. First, our sample cannot be considered representative of all Canadian older adults because we excluded those with no internet access and most of our respondents were White, English speaking, highly educated, and male. Respondents may have been in a more privileged position than the average Canadian in terms of decisions about housing, that is, they could hire private home care workers or pay for private residential care [72]. A selection bias may have occurred because people with higher eHealth literacy are more likely to subscribe to private panels such as this one [70,73]. Rhodes et al [74] point out the pitfalls of collecting data electronically without considering the “digital divide,” or with the inaccurate assumption that web access and use is equal among subgroups within a country’s population. Second, in the Leger panel the percentage of respondents from the Canadian territories was lower than the percentage from the provinces, whereas the percentage of Indigenous people is higher in the territories (eg, 86% in Nunavut) than in the provinces. Thus, it is possible that Indigenous people were not adequately represented in our sample. Compounding this limitation, only 43.3% of households in the First Nations reserves have access to high-speed internet [75]. Third, our sample was limited to older adults who had made the decision to move in the past few months or were planning to make this decision in the coming year. This criterion was somewhat restrictive, as, according to Leger Marketing, some respondents from the territories screened out at this point in the survey. Fourth, we were able to measure older adults’ intention to use eDA, but we cannot say that they will definitely use it. Studies that ask follow-up questions after a lapse of a few months can address this limitation. Finally, this study was conducted during the COVID-19 pandemic period. Although the pandemic affected housing decisions in older adults living in residential care [76], we did not include specific questions related to the pandemic; thus, our survey did not take this into account.

### Conclusions

Our study is the first to assess Canadian older adults’ intention to use an eDA to help them make housing decisions. This study makes both empirical and conceptual contributions to the field of eHealth behavior. We were able to provide a better understanding of the relationships between intention and its constructs and examine the effects of various variables on intention. In addition, we propose a modified parsimonious theoretical framework based on UTAUT, involving additional relevant concepts such as eHealth literacy. Research on older adults’ decision-making about housing (eg, eDA development, assessment of intention to use it, and eventually its implementation and integration into various care trajectories) has become increasingly relevant. This study is a step forward toward facilitating eDA implementation and integration initiatives. Our findings and conclusions can be applied in similar sociodemographic contexts where older people are an increasingly large proportion of the population and need support to play an active decision-making role throughout their care continuum.

## Appendix

Multimedia Appendix 1 Details of the conversion of the paper-based decision aid to electronic decision aid.

Multimedia Appendix 2 Consensus-Based Checklist for Reporting of Survey Studies.

Multimedia Appendix 3 Correlation matrix of the continuous variables (age, number of people in the household, eHealth literacy, performance expectancy, social influence, and facilitating conditions).

Multimedia Appendix 4 Factors associated with older adults’ intention to use the electronic decision aid in bivariate analyses.

## Abbreviations

CROSS: Checklist for Reporting of Survey Studies
DA: decision aid
DHLI: Digital Health Literacy Instrument
eDA: electronic decision aid
eHeals: Electronic Health Literacy Scale
SDM: shared decision-making
UTAUT: Unified Theory of Acceptance and Use of Technology

## Data Availability

The data sets used and analyzed during this study are available from the corresponding author upon reasonable request.
