# Supplementary material for: Canadian Older Adults’ Intention to Use an Electronic Decision Aid for Housing Decisions: Cross-sectional Web-Based Survey
Source: JMIR Aging. 2023 Jan 18;6:e43106. doi: 10.2196/43106 (PMC9947828; doi:10.2196/43106)
Supplement: Multimedia Appendix 1 [file aging_v6i1e43106_app1.docx]

Multimedia Appendix 1: Details of the conversion of the paper-based decision aid (DA) to electronic decision aid (eDA).

Briefly, we used the Qualtrics web-based platform and translated the existing paper-based DA content into electronic format. Thus, we retained the content of the paper-based DA. However, we updated the evidence about housing options to ensure that the eDA content was up to date with the latest evidence on housing decisions. This update of the evidence was performed using the methods published by Boland et al. (2017). Hence, we modified the few sentences that explain the following: to date, there is no robust evidence about the benefits and harms of specific housing options being superior to others. In addition, we added a definition of shared decision-making and information about the purpose of using the eDA on the first page. In the eDA, we added questions to know whether older adults were completing the eDA alone or with another person and their relationship to that possible other person.

We also replaced the evaluation to be completed by health care providers with reflection items to be completed by older adults and asked them about their motives for making the decision to move or to stay.

In the options section, we added an open option box: “Move to …*Please enter the location to which you have chosen to move*”.

Because older adults could complete the eDA with someone, we added a scale from 1 to 10 to rate how strongly they agree with the person who accompanied them during the process and suggested they revisit the decision with their relative if necessary.

Lastly, because the eDA can be completed without a health care provider, we recommended, at the end of the eDA, that older adults meet with their health care provider to discuss their health and needs and assess their independence.
