# Supplementary material for: Canadian Older Adults’ Intention to Use an Electronic Decision Aid for Housing Decisions: Cross-sectional Web-Based Survey
Source: JMIR Aging. 2023 Jan 18;6:e43106. doi: 10.2196/43106 (PMC9947828; doi:10.2196/43106)
Supplement: Multimedia Appendix 3 [file aging_v6i1e43106_app3.docx]

Multimedia Appendix 3: Correlation matrix of the continuous variables (age, number of people in the household, ehealth literacy, performance expectancy, social influence and facilitating conditions).

| **Pearson correlation coefficient, N = 1000** | | | | | | | | |
| --- | --- | --- | --- | --- | --- | --- | --- | --- |
|  | **Age** | **Number of people in the household** | **eHealth literacy (subjective)** | **eHealth literacy (objective)** | **Performance expectancy** | **Effort expectancy** | **Social influence** | **Facilitating conditions** |
| **Age** | 1.00 | -0.12 *P* <.001 | -0.05  *P =* .14 | -0.14  *P* <.001 | -0.01  *P* = .65 | -0.05  *P* = .13 | 0.0001  *P* = .10 | -0.04  *P* = .22 |
| **Number of people in the household** | -0.12 *P* <.001 | 1.00 | 0.03  *P* = .39 | -0.01  *P* = .69 | 0.07  *P* = .03 | -0.002  *P* = .96 | 0.08 *P* = .01 | 0.03  *P =* .36 |
| **eHealth literacy (subjective)** | -0.05  *P* = .14 | 0.03  *P* = .39 | 1.00 | 0.15  *P* <.001 | 0.18 *P* <.001 | 0.27  *P* <.001 | 0.18  *P* <.001 | 0.31 *P* <.001 |
| **eHealth literacy (objective)** | -0.14  *P* <.001 | -0.01  *P* = .69 | 0.15  *P* <.001 | 1.00 | 0.09  *P* = .002 | 0.30 *P* <.001 | 0.12  *P* <.001 | 0.27  *P* <.001 |
| **Performance expectancy** | -0.01  *P* = .65 | 0.07  *P* = .03 | 0.18  *P* <.001 | 0.09  *P* = .002 | 1.00 | 0.64  *P* <.001 | 0.75 *P* <.001 | 0.56  *P* <.001 |
| **Effort expectancy** | -0.05  *P* =.13 | -0.0017  *P* = .95 | 0.27  *P* <.001 | 0.30  *P* <.001 | 0.64  *P* <.001 | 1.00 | 0.58  *P* <.001 | 0.84  *P* <.001 |
| **Social influence** | 0.00011  *P* = .10 | 0.08  *P* = .01 | 0.18  *P* <.001 | 0.12  *P* <.001 | 0.75  *P* <.001 | 0.58  *P* <.001 | 1.00 | 0.56  *P* <.001 |
| **Facilitating conditions** | -0.04  *P =* .22 | 0.03  *P* = .36 | 0.31  *P* <.001 | 0.27  *P* <.001 | 0.56  *P* <.001 | 0.84  *P* <.001 | 0.56 *P* <.001 | 1.00 |
