# Supplementary material for: Canadian Older Adults’ Intention to Use an Electronic Decision Aid for Housing Decisions: Cross-sectional Web-Based Survey
Source: JMIR Aging. 2023 Jan 18;6:e43106. doi: 10.2196/43106 (PMC9947828; doi:10.2196/43106)
Supplement: Multimedia Appendix 4 [file aging_v6i1e43106_app4.docx]

Multimedia Appendix 4 : Factors associated with older adults’ intention to use the eDA from bivariate analyses.

| **Variable** | **Respondents**  **n (%)** | **β (95% CI)** | ***P > F*** |
| --- | --- | --- | --- |
| **Age** | 1000 (100) | -0.002 | .84 |
| **Sex** | 1000 (100) |  |  |
| Male (ref) | 548 (54.8) | - |  |
| Female | 452 (45.2) | .05 (-0.16; 0.26) | .63 |
| **Gender** | 1000 (100) |  |  |
| Male (ref) | 546 (54.6) | - |  |
| Female | 454 (45.4) | .11 (-0.15; 0.27) | .57 |
| **Level of education** | 995 (99.5) |  |  |
| A university certificate, diploma or degree (ref) | 420 (42.0) | - |  |
| A college, CEGEP or other non-university certificate or diploma | 264 (26.4) | .01 (-0.25; 0.28) | .91 |
| A high school (secondary school) diploma or equivalent, a registered apprenticeship or other trade certificate or diploma | 286 (28.6) | .01 (-0.24; 0.27) | .91 |
| Lower than a high school (secondary school) diploma or equivalent | 25 (2.5) | -0.16 (-0.85; 0.53) | .65 |
| **Province or territory of residence** | 1000 (100) |  |  |
| Ontario (ref) | 377 (37.7) | - |  |
| Quebec | 251 (25.1) | .49 (0.22; 0.76) | **<.001** |
| Western Canada | 295 (29.5) | -0.05 (-0.30; 0.2) | .71 |
| Eastern Canada | 77 (7.7) | -0.04 (-0.46; 0.37) | .83 |
| **Zone, based on postal code** | 991 (99.1) |  |  |
| Urban (ref) | 850 (85) | - |  |
| Rural | 141 (14.1) | -0.18 (-0.47; 0.11) | .22 |
| **Ethnicity** | 997 (99.7) |  |  |
| White (ref) | 906 (90.6) | - |  |
| Not white | 73 (7.3) | .02 (-0.38; 0.43) | .91 |
| Indigenous peoples of North America | 18 (1.8) | -0.52 (-1.32; 0.27) | .20 |
| **Marital status** | 999 (99.9) |  |  |
| Legally married (and not separated) (ref) | 516 (51.6) | - |  |
| Divorced | 152 (15.2) | -0.08 (-0.39; 0.22) | .59 |
| Widowed | 138 (13.8) | -0.08 (-0.40; 0.23) | .61 |
| Never legally married | 93 (9.3) | -0.16 (-0.53; 0.22) | .41 |
| In a common-law union | 81 (8.1) | .08 (-0.32; 0.48) | .70 |
| Separated, but still legally married | 19 (1.9) | .45 (-0.33; 1.23) | .25 |
| **Number of people in the household** | 1000 (100) | .1 | .13 |
| **Mother tongue** | 1000 (100) |  |  |
| English (ref) | 629 (62.9) | - |  |
| French | 283 (28.3) | .56 (0.32; 0.79) | **<.001** |
| Other | 88 (8.8) | .1 (-0.27; 0.48) | .59 |
| **Family income** | 1000 (100) |  |  |
| $100 000 or more (ref) | 172 (17.2) | - |  |
| From $75 000 to $99 999 | 153 (15.3) | .31 (-0.05; 0.68) | **.09** |
| From $50 000 to $74 999 | 221 (22.1) | .30 (-0.03; 0.65) | **.07** |
| From $25 000 to $49 999 | 262 (26.2) | .17 (-0.15; 0.50) | .29 |
| Less than $25 000 | 114 (11.4) | -0.14 (-0.54; 0.26) | .50 |
| I prefer not to answer | 78 (7.8) | -0.10 (-0.55; 0.35) | .66 |
| **eHealth literacy (subjective)** | 1000 (100) | .05 | **<.001** |
| **eHealth literacy (objective)** | 1000 (100) | .09 | **.04** |
| **Performance expectancy** | 1000 (100) | .93 | **<.001** |
| **Effort expectancy** | 1000 (100) | .75 | **<.001** |
| **Social influence** | 1000 (100) | .82 | **<.001** |
| **Facilitating conditions** | 1000 (100) | .76 | **<.001** |

The variables age, number of people in the household, e-Health literacy, performance expectancy, effort expectancy, facilitating conditions and social influence were analyzed as continuous variables.

The estimates β for each variable and its 95% CI are presented in the table.

Ref = reference category for the analysis.

Statistically significant associations are indicated in boldface.
